# A Rare Ventral Tongue Presentation of a Lingual Thyroglossal Duct Cyst in an Infant: A Case Report

**DOI:** 10.7759/cureus.106705

**Published:** 2026-04-09

**Authors:** Pinki Pargal, Pallavi Nigam, Preethi Anni Mercy Paul

**Affiliations:** 1 Plastic Surgery, Christian Medical College & Hospital, Ludhiana, IND; 2 Pathology, Christian Medical College & Hospital, Ludhiana, IND

**Keywords:** congenital cyst, excision, lingual cyst, midline oral cyst, thyroglossal duct cyst

## Abstract

Lingual thyroglossal duct cysts (TGDCs) represent the rarest form of thyroglossal duct cysts. We describe a four‑month‑old female who presented with a mid‑anterior ventral tongue swelling noted since birth. Magnetic resonance imaging (MRI) showed a well‑defined, non‑enhancing cystic lesion arising from the ventral surface of the tongue. Needle aspiration under sedation, followed by oral intubation and transoral excision, was performed without complications. Histopathology confirmed a thyroglossal cyst. The six‑month follow‑up demonstrated complete healing and no recurrence. Early recognition and definitive transoral excision yield excellent outcomes while avoiding airway compromise.

## Introduction

Thyroglossal duct cysts (TGDCs) are the most common congenital midline neck masses, occurring in approximately 7% of the population [1]. Lingual TGDCs are rare, comprising about 2% of all TGDCs and less than 1% of congenital tongue masses [2]. A recent infant cohort places their frequency between 0.6 % and 3 % of TGDCs [3]. Ventral tongue involvement is exceedingly uncommon and may present a diagnostic challenge due to its atypical location and nonspecific clinical features.

In infants, such lesions may remain asymptomatic initially, leading to delayed diagnosis until complications such as feeding difficulty, infection, or airway compromise arise. Additionally, ventral tongue cystic lesions can mimic other entities, including ranula, dermoid cyst, and lymphatic malformations, making accurate diagnosis essential for appropriate management. Imaging, particularly magnetic resonance imaging (MRI), plays a key role in delineating lesion characteristics, its anatomical extent, and its relationship to surrounding structures, thereby guiding surgical planning [4].

Given the rarity of ventral tongue TGDCs and the potential for diagnostic uncertainty, reporting such cases is important to improve clinical recognition and management. We present a rare case of a ventral tongue TGDC in a four-month-old infant, highlighting the diagnostic considerations and management approach.

## Case presentation

**Figure 1:**
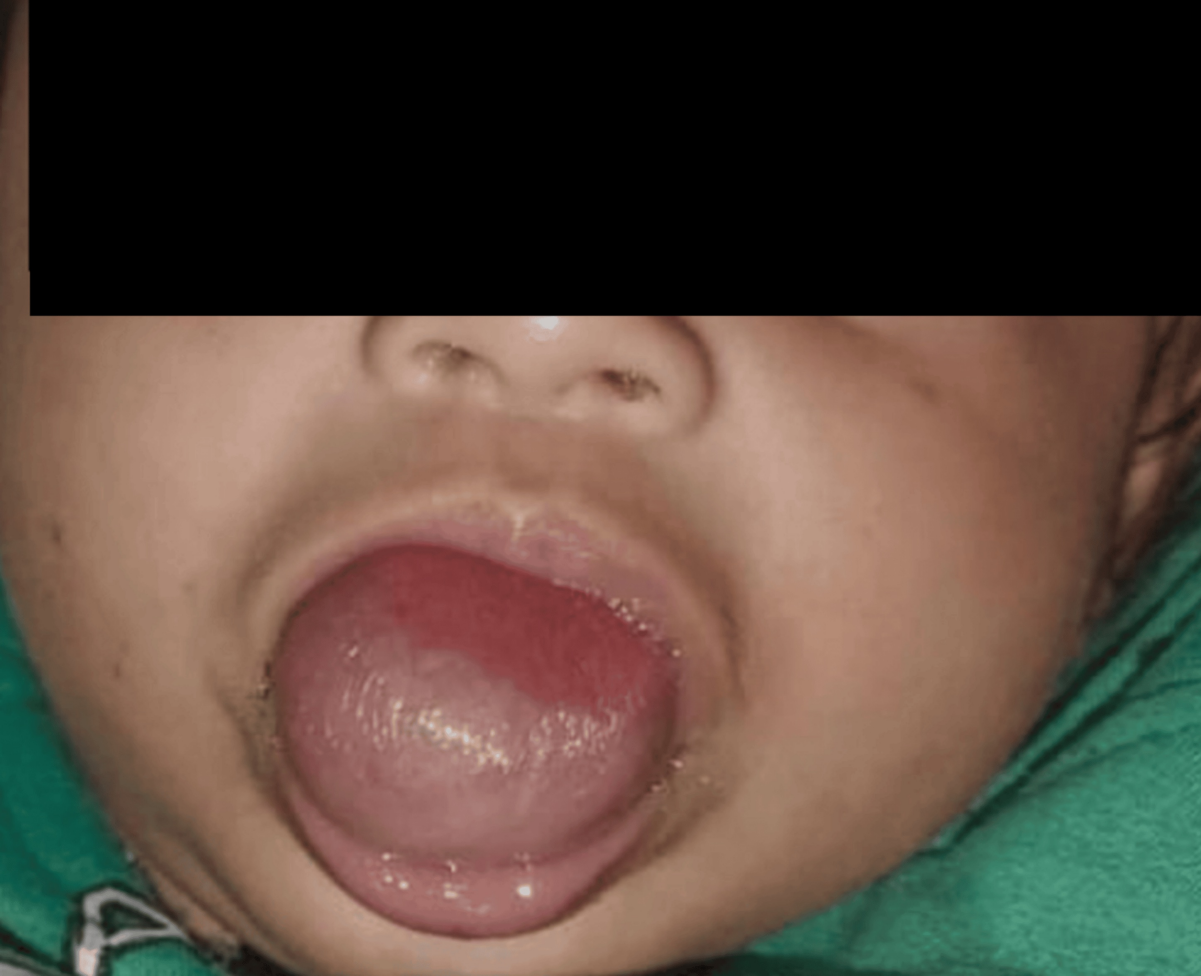
Preoperative picture showing cystic swelling involving the ventral surface of the tongue

A four‑month‑old female infant was brought by her parents for evaluation of a congenital swelling on the tongue. The swelling was first noticed at birth as a small midline bulge on the ventral surface of the anterior tongue and had shown slow, proportionate enlargement over time. There was no history of feeding difficulty, choking, failure to thrive, noisy breathing, stridor, cyanotic spells, drooling, oral bleeding, fever, or prior infection of the lesion. Crying and supine positioning did not precipitate respiratory symptoms. Clinical examination revealed a 4 × 2.5 × 2 cm, soft, cystic, fluctuant, translucent, midline mass occupying the anterior two-thirds of the ventral tongue (Figure 1). The overlying mucosa was intact, smooth, and of normal color, without ulceration, varicosities, or punctum. The thyroid gland was palpably normal and confirmed to be in situ by ultrasonography.

Investigations

**Figure 2:**
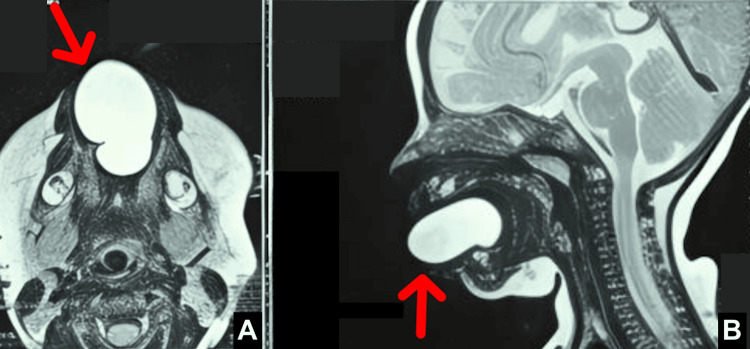
MRI showing a well-circumscribed, non-enhancing T1-hypointense/T2-hyperintense cystic lesion arising from the ventral surface of the tongue along its middle and anterior portion Magnetic resonance imaging of the oral cavity demonstrates a well-circumscribed, biloculated cystic lesion arising from the ventral aspect of the anterior tongue. (A) Axial T2-weighted image shows a markedly hyperintense biloculated lesion occupying the anterior oral cavity. (B) Sagittal T2-weighted image demonstrates anterior extension of the lesion beyond the labial folds, with significant mass effect on the dorsal surface and base of the tongue, resulting in posterosuperior displacement.

MRI demonstrated a well-circumscribed, non-enhancing, T1-hypointense, and T2-hyperintense cystic lesion arising from the ventral surface of the tongue along its mid and anterior portions (Figure 2). The lesion occupied almost the entire oral cavity and projected anteriorly beyond the labial folds, with an associated mass effect on the dorsal surface and base of the tongue, causing posterosuperior displacement. No internal enhancing solid component was identified. Thyroid ultrasonography confirmed a normally situated thyroid gland.

Differential diagnosis and perioperative planning

The clinical and imaging findings supported a diagnosis of a midline intralingual cyst. Differential considerations included TGDC, epidermoid or dermoid cyst, ranula, lymphatic malformation, mucous retention cyst, and foregut duplication cyst [5]. Given the midline location, positive transillumination, absence of floor-of-mouth involvement, and presence of an orthotopic thyroid gland, a working diagnosis of a lingual TGDC was made. Airway planning prioritized controlled conditions, with lesion decompression before intubation to preserve visualization and minimize trauma.

Procedure

**Figure 3:**
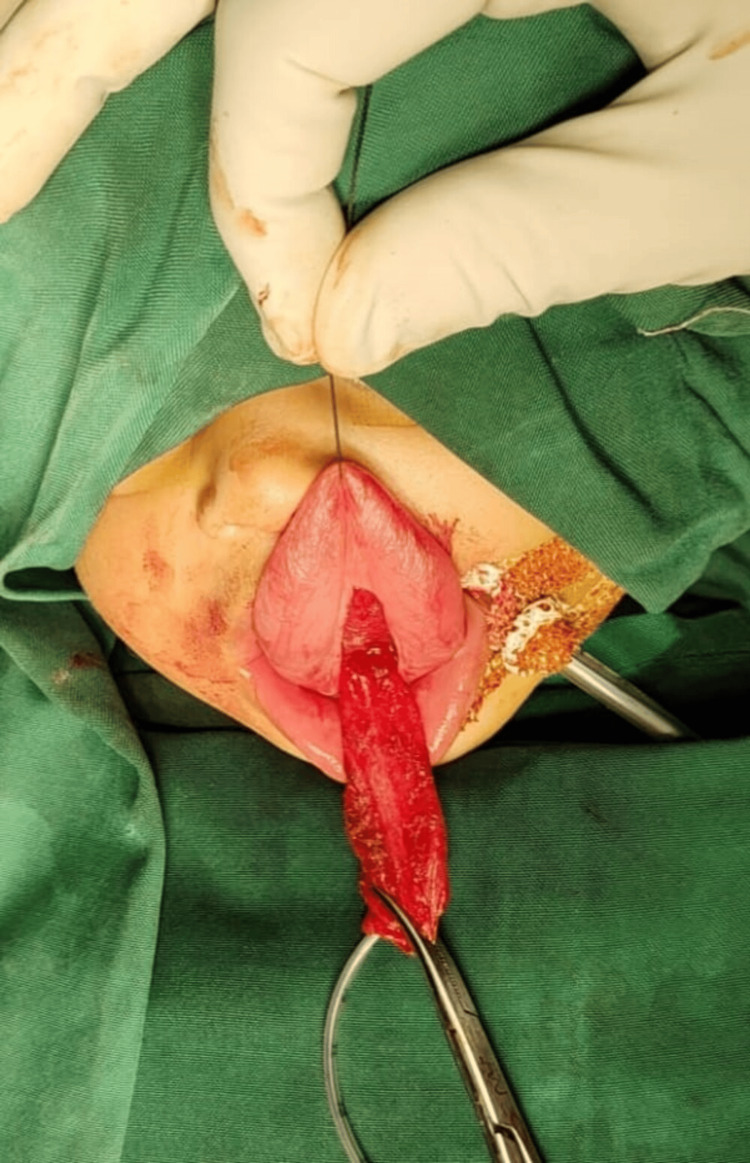
Intraoperative photo showing lingual cyst after aspiration

Under monitored intravenous sedation, the cyst was decompressed by sterile aspiration, yielding clear mucoid fluid and reducing its bulk. With improved tongue mobility and glottic visualization, the airway was secured by gentle orotracheal intubation. A transoral midline mucosal incision was placed directly over the decompressed cyst. Sharp and blunt dissection allowed circumferential mobilization along the capsule, maintaining an intact plane from the surrounding muscle fibers (Figure 3).

**Figure 4:**
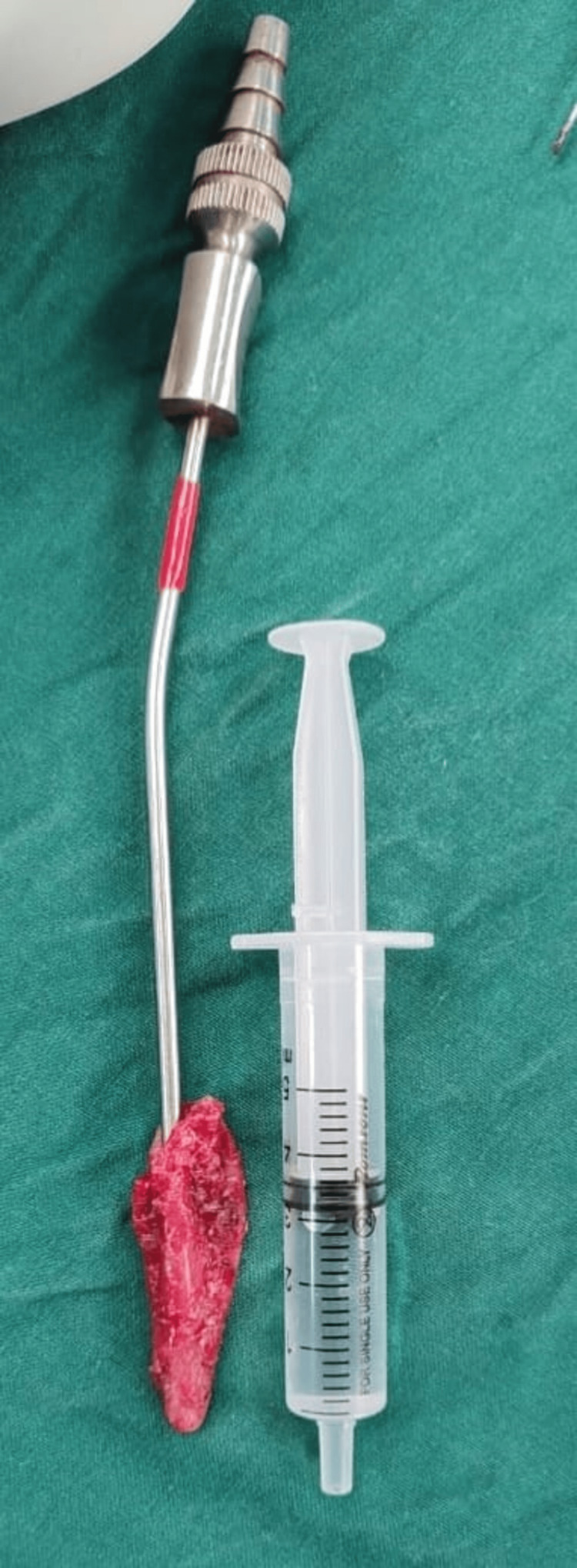
Excised cyst along with aspirated fluid

No tract extending to the foramen cecum or hyoid bone was identified. The cyst was excised en bloc (Figure 4).

**Figure 5:**
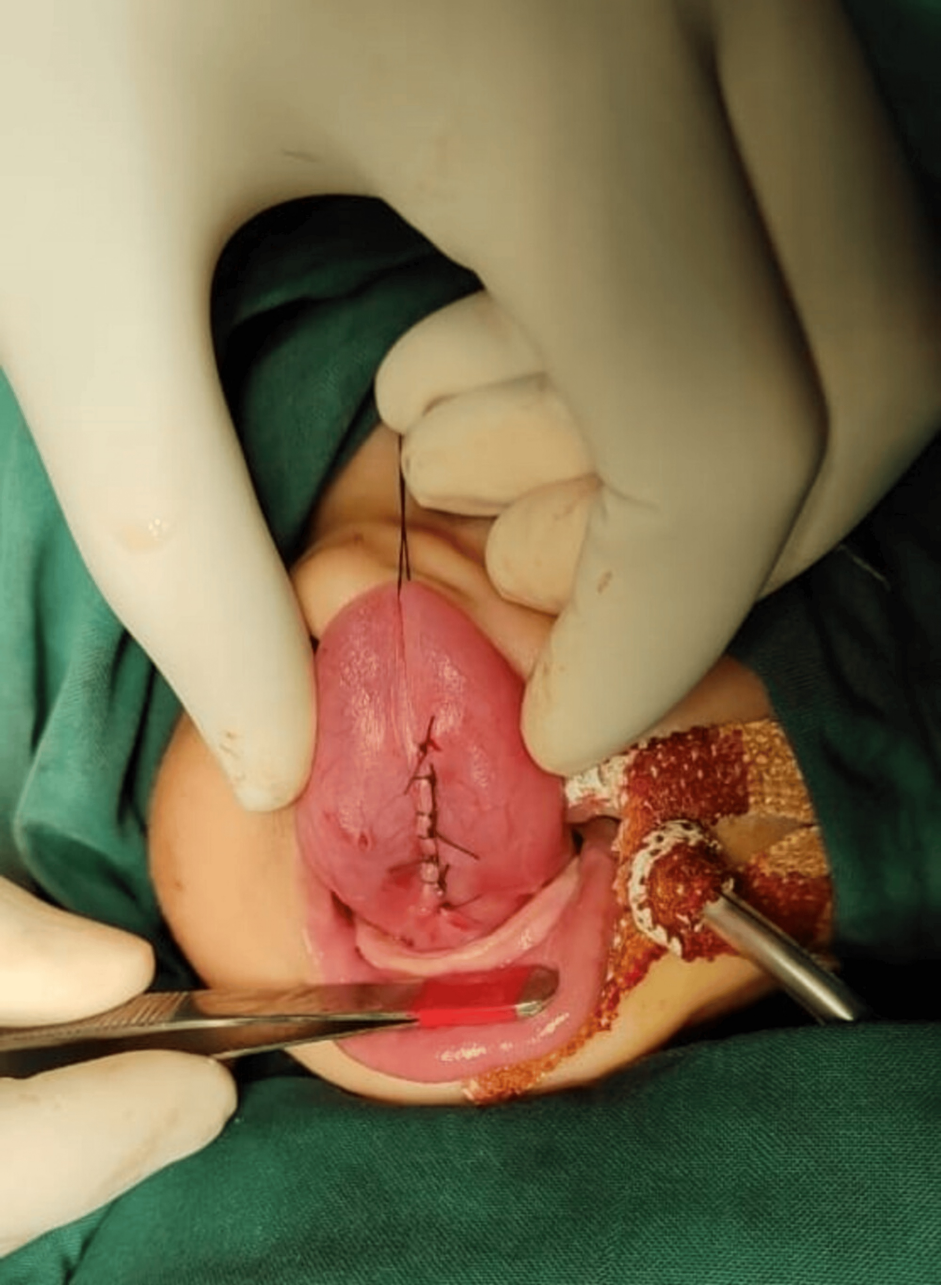
Postoperative photo after complete excision of the cyst and closure with absorbable sutures

Hemostasis was secured with bipolar cautery. The mucosa was closed with fine absorbable sutures (Figure 5). Extubation was uneventful.

Histopathology

**Figure 6:**
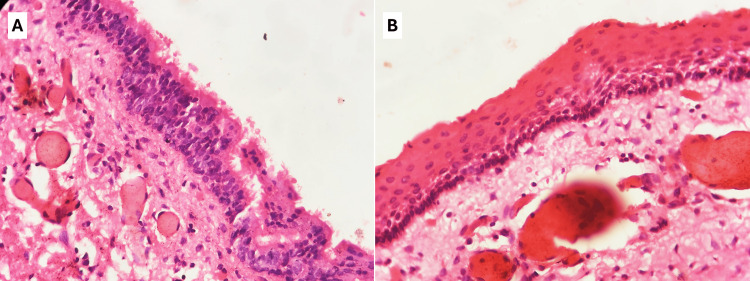
Thyroglossal duct cyst from the ventral surface of the tongue showing (A) lining of hyperplastic respiratory epithelium and (B) lining of stratified squamous epithelium. The cyst wall was comprised of delicate fibrocollagenous tissue with congested blood vessels and scattered mononuclear cell infiltrates. Hematoxylin and eosin, 400×.

Gross examination revealed a dark-brown cystic soft-tissue mass measuring 4 × 1.5 × 0.2 cm. The cut section showed the cyst to be thin-walled with an irregular inner lining. No papillary excrescences or nodularities were identified, grossly. Sections revealed a thin-walled cyst lined by stratified squamous epithelium, with focal areas showing hyperplastic respiratory epithelial lining. The wall consisted of delicate fibrocollagenous tissue with congested blood vessels. No thyroid tissue or neoplastic pathology was identified. The features were consistent with a TGDC (Figure 6).

Outcome and follow-up

**Figure 7:**
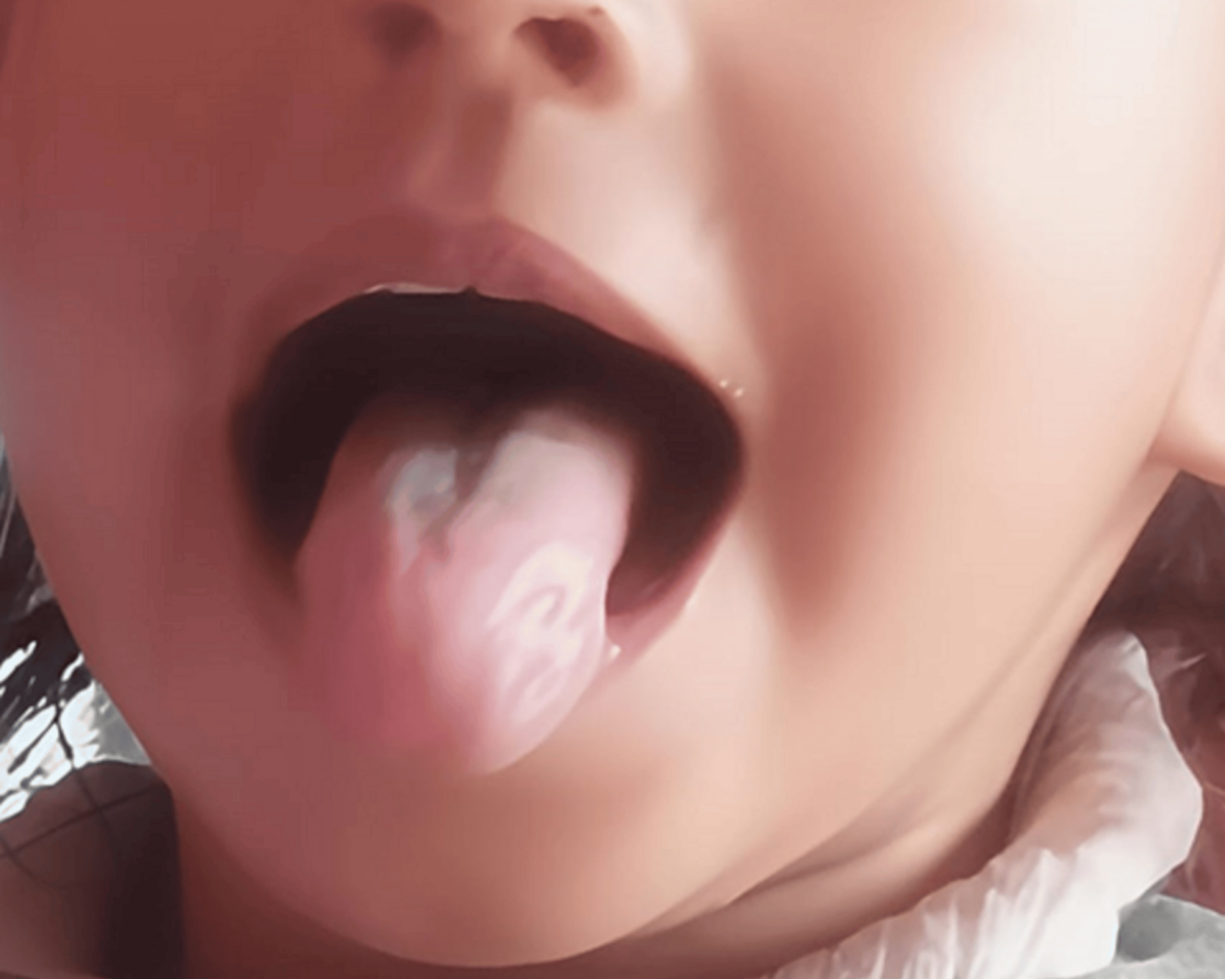
Six-month follow-up with no recurrence or complication

Feeds were resumed on postoperative day 1. The infant remained asymptomatic, and the six-month follow-up examination showed no recurrence or complications (Figure 7).

## Discussion

TGDCs arise from incomplete involution of the thyroglossal duct during embryological development, leaving epithelial remnants anywhere along the tract from the foramen cecum to the thyroid bed. Persistence of these remnants within the tongue is uncommon, and involvement of the ventral tongue is particularly rare, which explains the unusual presentation in this case [6]. Acute obstructive symptoms such as stridor, apnea, dysphagia, and respiratory distress are more commonly seen in lingual TGDC occupying the tongue base [7]. Imaging plays a key role in evaluation, with MRI typically demonstrating low signal intensity on T1-weighted images and high signal intensity on T2-weighted images, with absent or peripheral rim enhancement in uncomplicated cysts. These features help distinguish TGDCs from vascular malformations or solid masses [8].

The standard treatment for infrahyoid TGDCs is the Sistrunk procedure, which includes excision of the cyst along with the central portion of the hyoid bone and has been shown to reduce recurrence rates to less than 5%. However, for isolated intralingual TGDCs, several studies, including those utilizing endoscopic and robotic approaches, have demonstrated that complete transoral excision of the cyst without hyoid resection is effective and associated with lower morbidity [9]. Early surgical intervention in infants has been shown to yield favorable outcomes with low recurrence rates when appropriately managed [10]. In our case, transoral excision resulted in an uncomplicated postoperative course with no evidence of recurrence at six-month follow-up, supporting the adequacy of this approach for selected intralingual lesions.

This case highlights the importance of considering TGDC in the differential diagnosis of ventral tongue masses in infants, as early recognition and appropriate surgical management can prevent complications and lead to excellent clinical outcomes.

## Conclusions

Congenital mid‑anterior ventral‑tongue swellings, albeit uncommon, should prompt consideration of lingual TGDC. Confirmation of a normally located thyroid gland is a critical preoperative step. High‑resolution MRI confirms diagnosis and guides a minimally invasive transoral approach. Early definitive excision following pre‑intubation decompression to facilitate a controlled airway and meticulous transoral dissection to achieve complete removal while preserving tongue function achieves a cure with negligible morbidity and low recurrence risk.
